# Inverse Determination of Johnson–Cook Parameters of Additively Produced Anisotropic Maraging Steel

**DOI:** 10.3390/ma15010026

**Published:** 2021-12-21

**Authors:** Rocco Eisseler, Daniel Gutsche, Clemens Maucher, Hans-Christian Möhring

**Affiliations:** Institute for Machine Tools, University of Stuttgart, Holzgartenstrasse 17, 70174 Stuttgart, Germany; daniel.gutsche@ifw.uni-stuttgart.de (D.G.); clemens.maucher@ifw.uni-stuttgart.de (C.M.); hc.moehring@ifw.uni-stuttgart.de (H.-C.M.)

**Keywords:** additive manufacturing, machining simulation, inverse parameter identification, material property, maraging steel

## Abstract

In powder bed-based additive manufacturing (AM), complex geometries can be produced in a layer-wise approach. Results of material science experiments regarding material property identification, e.g., tensile strength, show interdependencies between the test load direction and the layer orientation. This goes hand-in-hand with the measured cutting force, changing with the relative angle between cutting direction and layer orientation in orthogonal cutting tests. However, due to the specific process characteristics, the layer orientation results in anisotropic material properties. Therefore, during machining, the material behaves depending on the buildup direction, which influences the cutting process. To predict this behavior, a simplified inverse approach is developed to determine the buildup direction-dependent parameters of a modified Johnson–Cook model for cutting simulation. To qualify these cutting models, mainly the cutting force and additionally the chip formation examined during orthogonal cuts are used. In the present paper, the influence of the laser-powder-bed-fusion (LPBF) process parameters on subtractive post-processing are shown. A good agreement between verification experiments and simulations is achieved.

## 1. Introduction

The LPBF process, as used for this research, utilizes lasers as a heat source to selectively melt powder in a thin layer. Repetition of this process over multiple layers can create complex three-dimensional structures. The layer-wise build-up material also leads to a rough surface that needs to be machined for most applications. For this machining, processes with a defined cutting edge are used in particular [1]. However, due to the selectively induced heat and large cooldown rates, a fine dendritic microstructure forms along the build-up direction [2]. This leads to an anisotropic material behavior, which also affects the cutting process [3].

The objective of machining simulation is to enable machining process optimization with a low expense of material- and time-intensive machining examinations. Suitable material models for machining simulation are often not available, especially for new materials. This also includes additively manufactured materials whose material properties are highly dependent on the manufacturing process. The aim of the work presented here is to model the influence of the build-up direction on chip formation in machining simulations and to provide adapted Johnson–Cook parameters for this purpose. The anisotropic behavior of specimens made from various steel and stainless steel alloys has been presented by numerous researchers. They all state a difference between build orientations. An almost linear decrease in ultimate tensile strength with an increase of build orientation φ is reported [4,5,6].

To take this influence into account in the machining simulation, inverse parameter identification can be used to determine the characteristic values of a material based on simulation calculations for a specific loading condition. In this way, the comparatively high strain rates and steep temperature gradients in machining processes can also be taken into account, which can only be mapped with great effort using experimental analyses. Simulation of machining processes is gaining more and more importance and is used both to get knowledge about basic machining mechanisms, as well as to optimize specific machining tasks [7,8]. The influence of a varied tool shape or of variable machining parameters on chip formation are exemplary subjects of intensive research work in recent years [9,10,11]. Furthermore, the machining temperature, residual stress states, and influences on microstructure formation were investigated by means of simulation [12,13,14]. In addition to the consideration of the machining process itself, the method of machining simulation has also been successively developed. The focus was, for example, on processes for remeshing and mesh refinement in the shear zone and approaches for the simulation of specific chip formation phenomena, such as shear chip formation [9,15]. However, the research topic was also the modeling of chip formation depending on the material as a function of its properties. Starting with differently conditioned low alloy steel [16], non-ferrous metals, such as titanium alloy Ti–6Al–4V [17] or nickel-based superalloys, such as Inconel 718 [18].

For the simulations to be conducted, the required material parameters, for instance Johnson–Cook parameters, usually are taken from the literature. There are mainly two approaches to determine the constants of the Johnson–Cook constitutive equation, which are used in the modeling of machining processes. The first approach is based on standardized experimental material tests, such as tensile and compression tests, or methods that reproduce the general conditions of machining processes, such as the split Hopkinson pressure bar (SHPB) test [19,20,21]. The searched individual Johnson–Cook constants are derived from the resulting flow curves using well-known mathematical methods [22]. A second approach, which is gaining in importance, uses the simulation of machining processes to get adapted material parameters [2,23,24,25,26,27,28,29]. Here, the material parameters are modified within chip formation simulations starting from available initial values while observing the influence on previously defined evaluation criteria. In this way, for example, the Johnson–Cook parameters are varied repeatedly within an increasingly decreasing range until the simulated criteria deviate only minimally from the experimentally determined criteria. The required initial parameters are taken from the literature or determined on the basis of material tests. Based on this approach, the orientation-dependent properties of additively produced M300 steel and their influence on chip formation in the orthogonal cut are to be used for the simulation-based determination of the Johnson–Cook parameters and validated based on experimentally determined comparison criteria. Here, both the cutting force and chip shape criteria are integrated.

## 2. Materials and Methods

### 2.1. Material

The here described inverse parameter identification was conducted on the example of additively manufactured specimens made of grade 300 maraging steel (short M300), according to ASTM A646. Manufacturers characterize the martensitic hot work tool steel as a material with both high hardness and high ductility. These properties enable the material with a high wear resistance and thus allow applications in mechanically and thermally highly stressed components, e.g., forming tools and molds. Additive manufacturing is a suitable method to create complex internal contours, such as channels for cooling fluids, in components like these. Table 1 shows the chemical composition of the material M300. Due to the increasing use of M300, obtaining detailed knowledge of both material properties [30,31,32] and behavior [33,34] under various conditions is a focus of ongoing research.

The Johnson–Cook constitutive equation [36] describing flow curves using few parameters in an easy-to-use manner is the basis of one of the most widely used material models. Equation (1) describes the von Mises flow stress with three terms considering different influences: the strain hardening, the strain rate, and the temperature.

(1) $$\sigma =\left[A+B\cdot \varepsilon^{n}\right]\cdot \left[1+C\cdot \ln{\dot{\varepsilon }}^{*}\right]\cdot \left[1-T^{*m}\right]$$

where

ε  Equivalent plastic strain
${\dot{\varepsilon }}^{*}=\frac{\dot{\varepsilon }}{{\dot{\varepsilon }}_{0}}$  Dimensionless plastic strain rate for ${\dot{\varepsilon }}_{0}=1.0\;s^{-1}$ and $T^{*}=0\;K$
$T^{*}=\frac{T-T_{r}}{T_{m}-T_{r}}$  Temperature ratio
$\left[A+B\cdot \varepsilon^{n}\right]$  Strain hardening for ${\dot{\varepsilon }}_{0}=1.0\;s^{-1}$ and $T^{*}=0\;K$
$\left[1+C\cdot \ln{\dot{\varepsilon }}^{*}\right]$  Influeence of strain rate on stress
$\left[1-T^{*m}\right]$  Influence of temperature on stress

with

(2) $${\left({\dot{\varepsilon }}^{*}\right)}^{\alpha }=1↔\alpha =0$$

(3) $$\left[D-ET^{*m}\right]=\left[1-T^{*m}\right]↔D=1\wedge E=1$$

(4) $$D=1=D_{0}^{k\cdot {\left(T-T_{b}\right)}^{\beta }}\Leftrightarrow k=0\wedge \beta \in \left\{ℜ\right\}\wedge T_{b}\in \left\{ℜ\right\}\wedge D_{0}\in \left\{ℜ/0\right\}$$

and choosing

(5) $$\beta =0\wedge T_{b}=0\wedge D_{0}=1$$

Equation (1) can be written in the generalized form:

(6) $$\sigma =\left[A+B\cdot \varepsilon^{n}\right]\cdot \left[1+C\cdot \ln{\dot{\varepsilon }}^{*}\right]\cdot {\left({\dot{\varepsilon }}^{*}\right)}^{\alpha }\cdot \left[D-ET^{*m}\right]$$

In the literature, Johnson–Cook parameters can be found for the most widely used materials. Usually, those values were determined experimentally with the known methods, e.g., based on tensile and compression tests, in some cases conducted under the thermal and mechanical conditions of machining processes. Unfortunately, it is challenging to find material models especially describing the behavior depending on the build-up orientation of additively manufactured parts. Table 2 shows the Johnson–Cook parameters of the above-mentioned maraging steel M300 used as material within this work. The data were found in the literature and has been elaborated from stress-strain curves for aged maraging steel [37].

### 2.2. Additive Manufacturing Process

**Figure 1:**
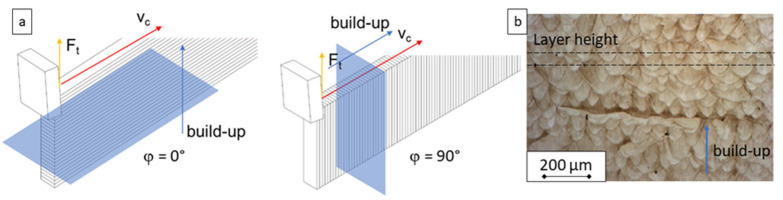
(**a**) Scheme of the built-up orientation angle φ; (**b**) microstructure of the utilized maraging steel [3].

The LPBF process was used within the described research work to create material specimens. It can be used to process a wide variety of metallic materials, such as aluminum alloys, steels, titanium alloys, and Ni-base super alloys. In theory, all weldable metallic materials are usable by means of the LPBF process [38]. The material specimens used here were produced on a RenAM 500Q LPBF system from Renishaw plc., Wotton-under-Edge, UK. It is equipped with four laser beam outputs, each with 500 W optical power, synchronously useable via 2-mirror galvanometers. The system has a build chamber with a built-up volume of 250 × 250 × 350 mm using a closed vacuum-assisted system for initial inertization and shielding gas recirculation (Renishaw plc., Wotton-under-Edge, UK). The specimens were produced with the inert gas nitrogen at a flow rate of 190 m^3^/h. The base plate was heated to 60 °C to reduce thermal influences on the support structures by gradually heating the build plate during the build-up. The layer height was set to 50 μm. The number of borders was set to two. The hatch distance was set to 75 μm. The hatch pattern was set to “stripes” with a length of 10 mm. The laser power was set to 150 W for each output for the borders and to 200 W for the hatching. The spot diameter of the machine was 80 μm. In this way, specimens with the shape of flat cuboids measuring 170 mm in length, 5 mm in thickness, and 25 mm in height were produced from the M300 powder. By orienting the specimens on the build platform, the build direction of the workpiece specimens was varied at angles of 0 and 90 degrees (Figure 1). The stated fine dendritic microstructure, which forms along the build-up direction, can be seen in Figure 1b. It is known that this layer-wise structure anisotropic material behavior affects the cutting process [3].

### 2.3. Machining Process

**Figure 2:**
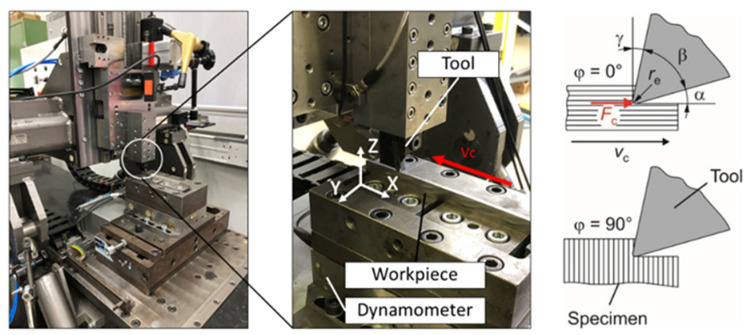
Test setup for experiments on orthogonal cutting and test conditions.

The specimens were used in experiments on orthogonal cutting on a test setup that enables a straight tool movement. The cuts were made both parallel (φ = 0 degrees) and normal (φ = 90 degrees) to the layer orientation regarding the long side of the specimen. Figure 2 shows the overall view of the test stand (left-hand side), as well as an exemplary specimen mounted on a dynamometer (Kistler Instrumente AG, Winterthur, Switzerland), an upstream run-in part, and the tool.

**Figure 3:**
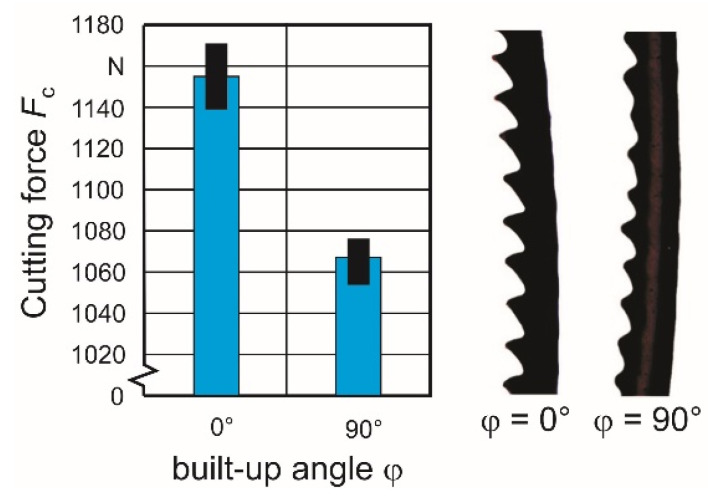
Experimental determined cutting force *F*_c_ and chip shape depending on material orientation.

The used tool insert is a parting and grooving tool type S229.0800.36 manufactured by Paul Horn GmbH, Tübingen, Germany. The inserts are made from a proprietary tungsten carbide compound called Ti 25. Table 3 lists essential tool characteristics. The experiments were conducted with a feed rate of *f* = 0.1 mm and a cutting speed of *v*_c_ = 80 m/min. For statistical validation, 30 replicate tests were conducted for both material orientations. Samples of the resulting chips were examined microscopically, and both the chip thickness *t* and the lamella distance *d* were determined in order to correlate them with the built-up angle φ. Figure 3 shows both the resulting mean value of the cutting force *F*_c_ of all individual tests, depending on the built-up orientation angle φ and sections of typical chips with a lamellar appearance. Mean values of the cutting forces were derived from the measured cutting force curves. The amplitude of the dynamic signal component is relatively low at less than 10% of the mean value. Due to the limited natural frequency of the dynamometer, it is not possible to derive any reliable quantified conclusions about the relationship between the dynamic component of the force curve and the lamella formation. The cutting force *F*_c_0_ = 1157.2 N in the case of φ = 0° and *F*_c_90_ = 1069.7 N with φ = 90° differ by 7.56%. This result corresponds with the above-mentioned context between tensile strength and built-up orientation φ.

### 2.4. Simulation

To obtain adapted Johnson–Cook parameters in regard to different built-up directions of the additively manufactured material, inverse parameter identification was implemented by means of the finite-element method. The goal of this procedure is to compare the results of chip formation simulations and machining experiments based on predefined criteria. In the iterative approach chosen here, a defined number of parameter sets was varied within progressively narrower limits and used as input variables for the simulations. In doing so, parameter sets were identified for which the simulation results approximate the experimentally determined criteria as closely as possible. The limits of the next iteration step result from those two parameter sets with the best approximation. The starting parameter range of the first iteration step was created by means of a DoE on the basis of the Latin hyper-cube method. Due to the approach commonly described in the literature, the cutting force *F*_c_ was taken into account in the first simulation runs [39,40]. The two-dimensional simulation model was developed using DEFORM 3D™ V12.01 (V12.01, Scientific Forming Technologies Corp., Columbus, OH, USA). The shape of the previously mentioned turning tool has been taken into account by the clearance angle α = 12° and the rake angle γ = 15°, as well as the cutting-edge radius *r*_b_ = 47.6 µm. The part’s mesh is generated with local refining and a maximum mesh number of 10,000. Focusing on the Johnson–Cook parameters responsible for the strain hardening effect, sets with varied parameters A, B and n based on Table 2 were prepared in a design-of-experiment approach, which is based on the Latin hypercube method. The upper and lower limits of the parameter ranges shown in Table 4 were determined to be +20% and −20% of the values described in the literature.

**Figure 4:**
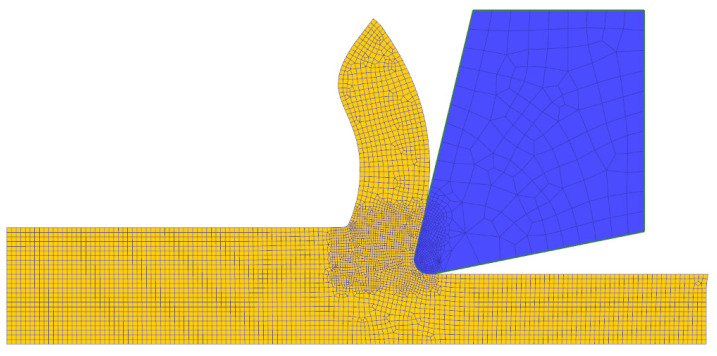
FEM-based model and resulting flow chip formation using the start parameters in the first identification iteration.

The parameters C and m remained unchanged during the simulation phase. In a first iteration, a number of 100 individual parameter sets were determined within the limits. To achieve an even cutting force progression, the initial simulations were conducted without any damage criterion resulting in the formation of flow chips. This simplification was also made with regard to the cutting force measured and subsequently averaged in the experimental investigations and the fact that the dynamic force component cannot be unambiguously quantified and thus used as a comparison criterion. The used simulation software prefers the generalized form of the Johnson–Cook material model. Figure 4 shows the 2D simulation model with the meshed part and tool, as well as the resulting chip shape using the start parameters.

## 3. Results and Discussion

**Figure 5:**
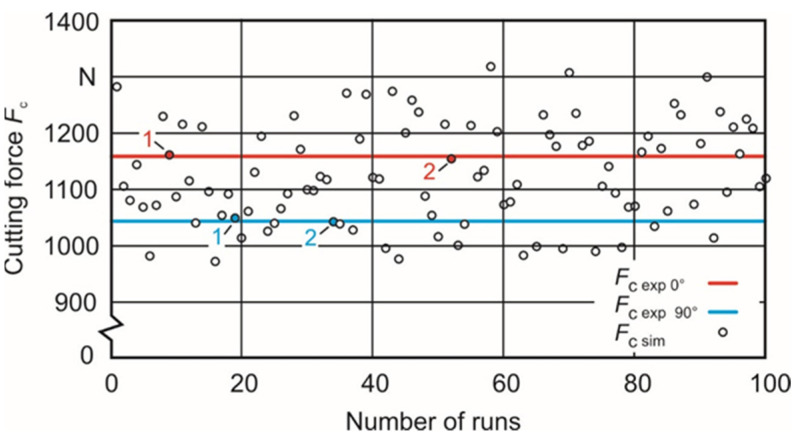
First iteration with 100 runs between the upper and lower limit and experimentally gained cutting forces for orientations φ = 0° and 90°.

Figure 5 shows the correlation between the cutting force value and the number of the individual parameter sets resulting from the first iteration. The experimentally determined mean values of the cutting forces based on the two built-up orientations, φ = 0° and 90°, are represented by a red (*F*_c_0_ = 1157 N) and a blue (*F*_c_90_ = 1067 N) line. Two parameter sets with the best cutting force approximation for each material orientation (set 0_1 and set 0_2, set 90_1 and 90_2) were determined.

**Figure 6:**
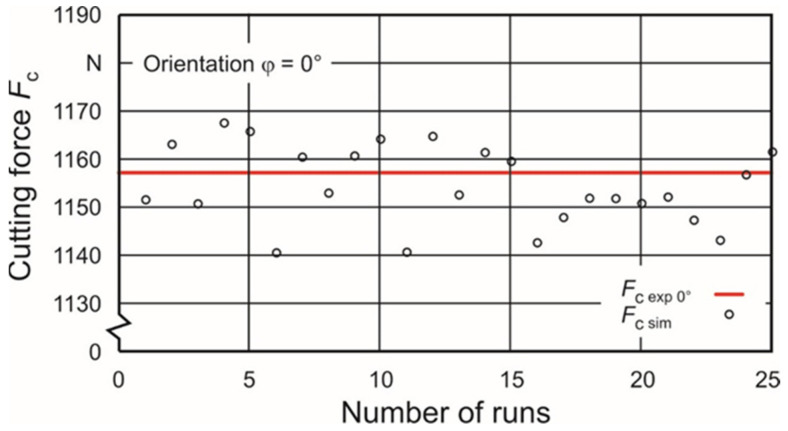
Second iteration with 25 runs for orientation φ = 0°.

**Figure 7:**
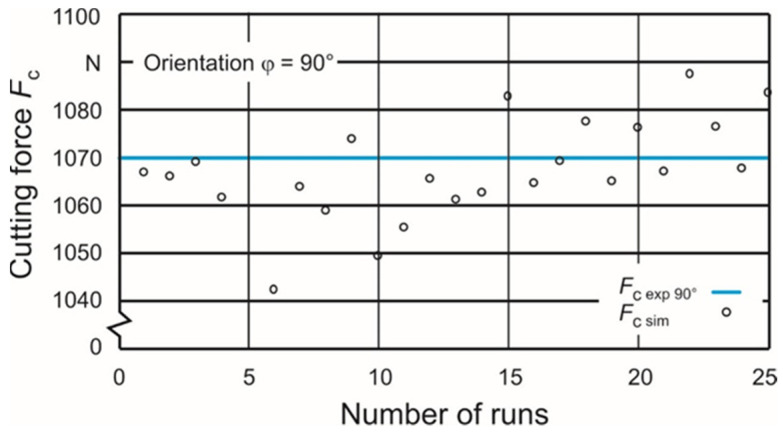
Second iteration with 25 runs for orientation φ = 90°.

These sets are used as upper and lower limits for a second identification iteration to get closer to the experimentally gained cutting force values. The nearest approximation can be reached with the parameter sets #9 and #52 in the case of the orientation angle φ = 0° and with the sets #7 and #64 in the case of φ = 0°. These parameter sets (Table 5) were used as new limits for the second identification iteration based on a DOE with 25 runs each. Figure 6 and Figure 7 show the calculated cutting forces depending on the individual sets. A renewed search for the parameter sets with the closest approximation to the experimentally gained cutting force values leads to sets #15 and #24 in the case of the orientation angle φ = 0° and sets #3 and #4 in the case of φ = 90°. The belonging Johnson–Cook parameters A, B and n are shown in Table 6.

**Figure 8:**
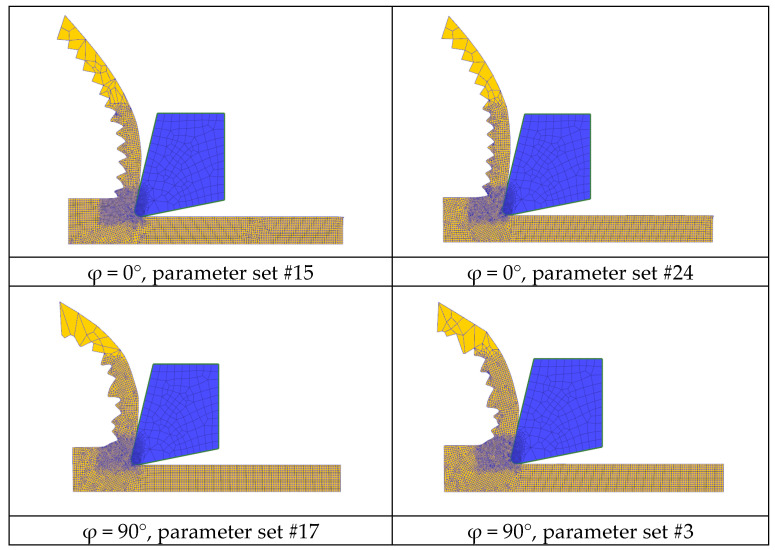
Simulation results depending on the built-up angle φ and different parameter sets.

To validate these final results and to gain information about the difference between them influencing the chip formation, the chip shape was taken into account as further criteria (Figure 8). Consequently, both the mean chip thickness *t* and the mean distance *d* between the edge of two neighboring chip lamellas was determined on experimentally produced chips, as well as on chips resulting from simulations.

Regarding this, the previously mentioned FE model was extended with a fracture model according to Cockroft and Latham [41] to enable the formation of lamella chips. Figure 8 shows the simulation results, where the chip formation depending on the chosen parameter set could be compared. It is noteworthy that chips resulting from the build-up angle φ = 0° show a more regular succession of the individual lamellas than those with φ = 90°. In addition, the chip shapes are little slimmer under condition φ = 0°. In the case of the direct comparison of different sets at the same built-up angle φ = 0°, it is noticeable that the lamellas originating from set #15 take a larger share of the total chip width than those from set #24.

**Figure 9:**
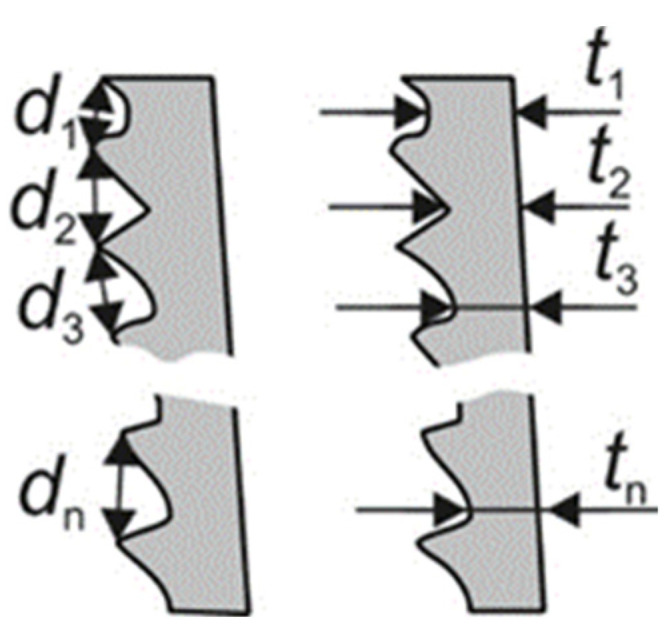
Chip shape criteria chip thickness *t* and lamella distance *d*.

To obtain information about the correlations between chip thickness *t* as well as lamella distance *d* and the parameter sets, several measurements were conducted on chips gained in both chip formation simulations and cutting experiments. The chip thickness was determined, regarding Figure 9, always at the thinnest point between the individual lamellas. The lamella distance was measured from edge to edge.

**Figure 10:**
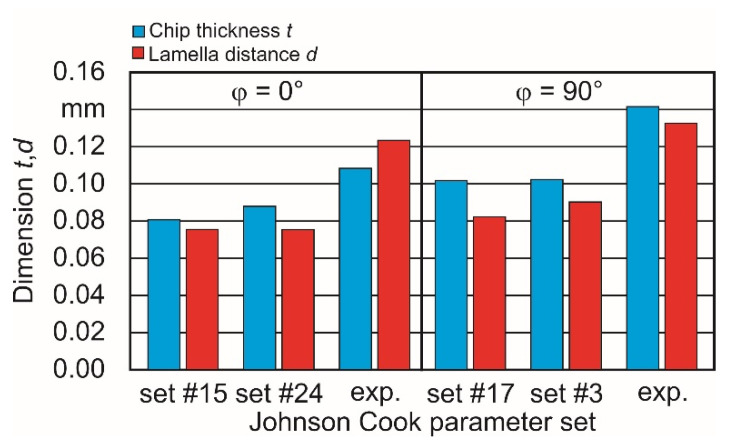
Chip shape criteria *t* and *d* depending on model and experiment-based results.

Figure 10 shows that both the mean values of the simulated chip thickness and the lamella distance are below the mean values of the experimentally gained chips. Table 7 shows the relative deviations between the individual criteria of the simulated chip shapes and those of the experimentally obtained ones.

**Figure 11:**
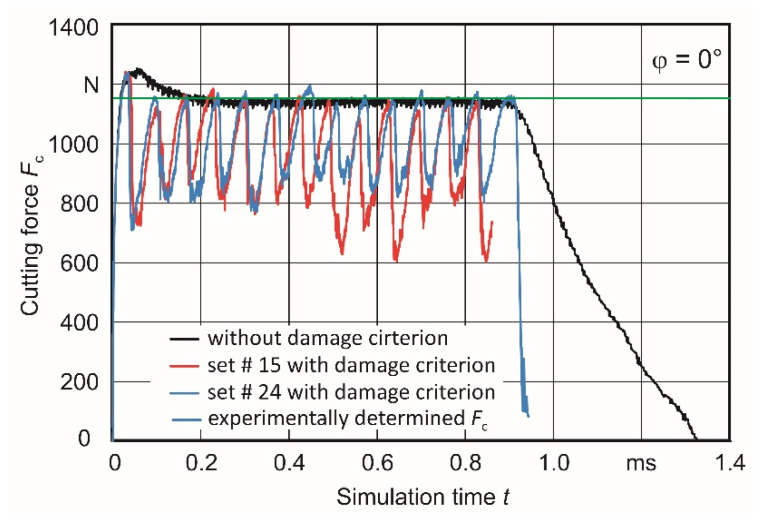
Comparison of simulation-based determined (both with and without damage criterion) and experimentally gained cutting forces *F*_c_ for φ = 0°.

The variable cutting forces determined with the damage criterion are on average on a lower level than the cutting force with pure flow chip formation (without damage criterion). The maximum values always correspond to the cutting force with flow-chip formation when the individual lamellae reach their maximum thickness. Figure 11 illustrates this for the case of the built-up angle φ = 0° for the parameter sets #15 and #24.

Overall, it can be seen that the dependence of the criteria on the built-up angle can be mapped in the trend. However, it should also be mentioned that the inclusion of further comparison criteria, in particular the dynamic cutting force component but also, for example, the machining temperature, seems advisable for further minimization of the present deviations.

## 4. Summary and Conclusions

The objective of this paper was to present inverse parameter identification based on FE simulations to determine the Johnson–Cook parameters A, B, and n for maraging steel M300 used for additive manufacturing in the two built-up directions.

- For a later comparison of experimentally and simulatively obtained criteria, maraging steel M300 specimens were prepared with both build-up directions, φ = 0° and 90°, by using the LPBF Process. In machining experiments, both the cutting force Fc and the chip shape characteristic were determined. The cutting force depends on the built-up orientation and differs by 7.56%.
- The required 2D finite element model was prepared using DEFORM 3D™ V12.01 and used to get sets of A, B and n Johnson–Cook parameters, which lead to the closest approximation to the experimental cutting forces. Start parameters to set up a DOE were taken from the literature.
- As additional criteria, both the chip thickness and the lamella distance of experimentally and simulative gained chips were determined and compared. While the absolute differences between the criteria of the experimentally and simulatively obtained chip properties are comparatively large, the dependence of both the chip thickness and the lamella distance on the buildup direction can be confirmed in the trend. The closest results are 26.6% difference of the chip thickness between φ = 0° and 90° in the simulation and 30.6% in the experiment. For the case of the lamella distance, the closest approximation has been reached with 9.3% in the simulation compared with 7.3% in the experiment.
- With reference to the simplified approach used in this work, further cutting experiments with a high-resolution and high-sensing force measurement system are planned. In this context, not only the influence of the built-up angle φ, but also the influence of individual layers in their interaction with lamellar chip formation is to be considered. In addition, temperature measurements in the area of the cutting zone are to be included. With additional criteria derived from this, further simulations are then to be performed with regard to a more accurate inverse parameter identification.

## Figures and Tables

**Table 1:** Chemical Formulation of Maraging Steel M300 [35].

Alloy Element	C	Cr	Mn	P	S	Si	Ti	Mo	Ni
Min. alloy content [%]							0.8	4.5	17
Max. alloy content [%]	0.03	0.25	0.15	0.1	0.1	0.05	1.2	5.2	19.0

**Table 2:** Johnson–Cook Parameters for Maraging Steel M300 According To [37].

A	B	n	C	M
758.0 MPa	172.147 MPa	0.2258	0.0522	0.7799

**Table 3:** Tool Specifications.

Tool Feature	Value
Rake angle γ	15°
Wedge angle β	63°
Clearance angle α	12°
Cutting edge radius *r*_e_	47.6 µm

**Table 4:** Parameter Range Limits for The First Identification Iteration Regarding Start Parameters from The Literature.

Johnson–Cook Parameter	A (MPa)	B (MPa)	n ()
Start values from literature	758.0	172.147	0.2258
**Iteration 1**			
Upper limit (+20%)	910.108	206.576	0.0626
Lower limit (−20%)	606.738	137.718	0.0418

**Table 5:** Second Iteration With 25 Runs Each Between the Upper and Lower Limit for Orientation Angles φ = 0° and 90°.

J.–C. Parameter	A (MPa)	ps#	B (MPa)	ps#	n ()	ps#
**φ = 0° Iteration 2**						
Upper limit	818.093	52	170.276	9	0.2269	52
Lower limit	809.121	9	154.213	52	0.1898	9
**φ = 90° Iteration 2**						
Upper limit	709.990	7	204.301	64	0.2140	64
Lower limit	657.995	64	174.550	7	0.2098	7

**Table 6:** Resulting Johnson–Cook Parameters A, B, and n After the Second Identification Iteration.

J.–C. Parameter	ps#	A (MPa)	B (MPa)	n ()
**Built-up angle φ = 0°**				
First closest	15	816.720	163.850	0.2166
Second closest	24	809.234	167.667	0.2027
**Built-up angle φ = 90°**				
First closest	17	705.004	179.962	0.2130
Second closest	3	660.754	203.009	0.2112

**Table 7:** Chip Shape Criteria T and D Depending on Model and Experiment-Based Results.

Compared Results of	Difference in Chip Thickness *t* (%)	Difference in Lamella Distance *d* (%)
φ = 0° set# 15 to φ = 0° exp.	33.8	63.0
φ = 0° set# 24 to φ = 0° exp.	22.9	63.2
φ = 90° set# 17 to φ = 90° exp.	38.4	60.1
φ = 90° set# 3 to φ = 90° exp.	38.7	47.6
φ = 0° set# 15 to φ = 90° set 17	26.2	9.3
φ = 0° set# 24 to φ = 90° set 3	15.7	18.7
φ = 0° exp. to φ = 90° exp.	30.6	7.3

## Data Availability

Not applicable.
